# Mitochondrial Biomarkers Reflect Semen Quality: Results from the MARCHS Study in Chongqing, China

**DOI:** 10.1371/journal.pone.0168823

**Published:** 2016-12-22

**Authors:** Guowei Zhang, Zhi Wang, Xi Ling, Peng Zou, Huan Yang, Qing Chen, Niya Zhou, Lei Sun, Jianfang Gao, Ziyuan Zhou, Jia Cao, Lin Ao

**Affiliations:** 1 Department of Environmental Health, College of Preventive Medicine, Third Military Medical University, Chongqing, China; 2 Institute of Toxicology, College of Preventive Medicine, Third Military Medical University, Chongqing, China; Kunming Institute of Zoology, Chinese Academy of Sciences, CHINA

## Abstract

Unexplained infertility requires that more sensitive and mechanism-based biomarkers should be developed and used independently of or in addition to conventional semen parameters for an infertility diagnosis. In the present study, semen samples were collected from young men participating in the Male Reproductive Health in Chongqing College students (MARCHS) cohort study in the follow-up stage in 2014. Conventional semen parameters were measured in all 656 participants, whereas sperm mitochondrial membrane potential (MMP), mitochondrial DNA copy number (mtDNAcn), mtDNA integrity and apoptotic parameters were measured among 627, 386, 362, and 628 participants, respectively. We found that sperm MMP was significantly positively correlated with all of conventional semen parameters including semen volume (r = 0.090, p = 0.025), sperm concentration (r = 0.301, p<0.01), total sperm count (r = 0.324, p<0.01), and progressive motility (r = 0.399, p<0.01); sperm MMP was also negatively correlated with Annexin V^+^ sperm (r = -0.553, p<0.01); mtDNAcn was significantly negatively correlated with sperm concentration (r = -0.214, p<0.01), total sperm count (r = -0.232, p<0.01), and progressive motility (r = -0.164, p = 0.01); mtDNA integrity was also significantly positively correlated with sperm concentration (r = 0.195, p<0.01), total sperm count (r = 0.185, p<0.01), and progressive motility (r = 0.106, p = 0.043). After adjusting for potential confounders, these relationships remained significant. Furthermore, we explored the potential effects of lifestyles on such mitochondrial biomarkers and found that the current drinkers displayed a higher level of sperm MMP; additionally, mt DNAcn was increased with age. The results indicated that certain mitochondrial biomarkers could serve as predictors of semen quality in a general population, and the study provides a baseline for the effects of population characteristics and lifestyles on such mitochondrial markers.

## Introduction

Approximately 10–15% of couples of reproductive age are infertile [1–3]. Male factors in particular account for 50% of these cases [4]. Traditionally, semen parameter analyses recommended by WHO have been used to diagnose the male semen quality, but these analyses may still fail to detect subtle sperm defects present in patients with male factor infertility [5]. Moreover, accumulating evidence has linked exposure to environmental pollutants and undesirable lifestyles to male reproductive health [6–9]. However, uncertainties remain about the effects of environmental exposure and lifestyle on semen parameters in the general population because the exposure to these factors is so low. Indeed, our previous studies revealed that polycyclic aromatic hydrocarbons (PAHs) or phthalate esters (PAEs) exposure at the environmental level was not associated with conventional semen parameters or morphology [10–11]. In this context, more sensitive and mechanism-based biomarkers should be developed and used independently of or in addition to conventional semen parameters for the infertility diagnosis or in the risk-assessment process.

Mitochondria in sperm are factories for energy production via oxidative phosphorylation (OXPHOS), namely ATP synthesis. ATP synthesis requires a chemiosmotic proton gradient across the inner mitochondrial membrane, the major electric component of which is usually translated into mitochondrial membrane potential (MMP) [12]. MMP is also necessary to sequester calcium ions and maintain calcium homoeostasis. Thus, MMP is an important indicator of mitochondrial function. Because mitochondrial DNA (mtDNA) encodes 13 subunits of the enzymatic complexes in the OXPHOS, any genetic variation of mtDNA will directly affect energy production. MtDNA copy number (mtDNAcn) and mtDNA integrity are two major mitochondrial genetic features. Because of the lack of protective histones and DNA repair capacity, mtDNA is particularly vulnerable to damage factors, such as reactive oxygen species (ROS) or adducts [13–14]. Therefore, mitochondrial markers may be sensitive predictors of semen quality damage.

Indeed, the search for more optimal predictors of semen quality has resulted in a growing focus on these mitochondrial markers. MMP was found to be associated with conventional semen parameters such as sperm motility [15], and the reduction in sperm MMP has been regarded as an early apoptotic event [16]. mtDNA content was found to increase in human sperm collected from abnormal semen samples [17]. Moreover, different mtDNA copies per cell in progressive and non-progressive human spermatozoa were also observed [18]. Song et al. [19] further demonstrated a significant increase in mtDNAcn and a decrease in the mtDNA integrity of the sperm samples from patients with abnormal semen parameters. Nonetheless, the number of such studies concerning associations between these mitochondrial biomarkers and conventional semen parameters remain limited. Furthermore, most of these related studies recruited participants from infertility clinic, not from general populations, which may increase the possibility of a selection bias.

The present study was a portion of the Male Reproductive Health in Chongqing College Students (MARHCS) cohort study established in 2013, which aimed to investigate the potential effects of environmental and socio-psycho-behavioral factors on male semen quality in a general population. The objective of the present study was to investigate potential associations between mitochondrial biomarkers (MMP, mtDNAcn, and mtDNA integrity) and those classic indicators of semen quality including conventional semen parameters and apoptotic parameters, and to observe whether these mitochondrial biomarkers can be regarded as predictors of semen quality in the general study population. Furthermore, we explored the effects of population characteristics and lifestyles on such mitochondrial markers.

## Materials and Methods

### Study population and questionnaire

The present study was performed using data collected in 2014 from male participants in the MARHCS study, a prospective cohort study conducted since June 2013. This study was approved by the Ethics Committees of the Third Military Medical University, and written informed consent was obtained from each volunteer. The study included collection and examinations of blood, urine, and semen samples; a detailed social-physical-behavioral questionnaire; and a physical examination. Individuals were excluded if they met any of the following criteria: <18 years old; <2 or >7 days of abstinence; a history of inflammation of the urogenital system, epididymitis or testicular injury; a history of incomplete orchiocatabasis; a history of varicocele treatment; absence of the pubis, prominentia laryngea or testis; abnormal breasts or penis; varicocele; or epididymal knob. A total of 666 eligible subjects attended the followed-up procedure in 2014, among which 10 failed to provide semen sample. More detailed information about the study design, screening process and lifestyles questionnaire can be found in the previous report [20].

### Semen collection and analyses

As described previously [20], the semen samples were collected in a special semen container via masturbation in a private room. Upon receipt, the semen appearance was recorded, and semen volume was measured by weighing, assuming 1 g of weight was equal to 1 ml of volume. Then, the samples underwent liquefaction at 37°C in a water bath. Conventional semen parameters, including sperm concentration, total sperm count, and motility, were analyzed using computer-aided sperm analysis (CASA) according to the WHO guidelines (2010).

To reduce the variation of the assessment of sperm characteristics, one well-trained technician performed the routine semen analyses throughout the study. All of the samples were analyzed within 60 min of collection. For the assessment of sperm morphology, two fresh semen smears were made, air-dried, fixed, and stained using the method described in the 2010 WHO manual.

### MMP determination

MMP was measured using 5,5’,6,6’,tetrachloro-1,1’,3, 3’-tetraethylbenzimidazolcarbocyanine iodide (JC-1; Sigma-Aldrich Chemical Co., USA), a lipophilic cationic dye. JC-1 was dissolved in dimethyl sulfoxide (DMSO). After removing the seminal fluid, aliquots of 2×10^6^ sperm cells were incubated in 1 ml of PBS containing 5 μg/ml of JC-1 for 20 min at 37°C in the dark. Then, the sperm cells were rinsed twice with PBS and analyzed using a flow cytometer (Accuri C6, BD Biosciences, San Jose, CA). For sperm with high MMP, JC-1 spontaneously forms J-aggregates with intense red fluorescence, which is measured in the FL2 channel. For sperm with low MMP, JC-1 remains in the monomer form and exhibits green fluorescence, which is measured in the FL1 channel. The value was presented as the percentage of high MMP cells.

### mtDNAcn analysis

The total DNA was extracted from each semen sample using the E.Z.N.A.^TM^ DNA Isolation Kit (Omega Bio-tek Inc, Norcross, GA, USA) according to the manufacturer’s instructions. The mtDNAcn analysis was based on the ratio of a mitochondrial gene (16S rRNA) to a nuclear gene (GAPDH) [19]. The quantitative real-time polymerase chain reaction (Q-PCR) assay was performed in the CFX system (Bio-Rad, Hercules, CA) using SYBR^®^ Premix Ex Taq^TM^ II (Takara, Japan). The sequences of primers were as follow: 16S rRNA forward: 5’-ACTTTGCAAGGAGAGCCAAA-3’ and reverse: 5’-TGGACAACCAGCTATCACCA-3’; GAPDH forward: 5’-GGATGATGTTCTGGAAGAGCC-3’ and reverse: 5’-AACAGCCTCAAGATCATCAGC-3’. The PCR reactions were performed under standard conditions using 10 ng of total DNA template in a 25 μl volume with 50 cycles of a denaturation step of 15 s at 95°C and a hybridization and extension step of 15 s at 60°C. A melting curve analysis was used to verify the accuracy of the target amplification. A standard DNA sample was obtained by pooling DNA from 20 participants randomly selected from the population and was used to adjust the difference among tests.

### mtDNA integrity analysis using long PCR

To investigate the mtDNA integrity in human sperm cells, half of the mitochondrial genome (8.7 kb) was amplified using long PCR as described previously [19]. The 8.7-kb fragment contains the common deletion types (7.4 and 4.9 kb) and several genes encoding subunits of respiratory complexes. The long PCR was performed with 200 ng of sperm DNA using *TaKaRa LA Taq*^*®*^ (Takara, Japan) in a 50 μl volume: 1×LA Taq buffer, 10 pmol of each primer (5’-AAGGATCCTCTAGAGCCCACTGTAAAG-3’ and 5’-TTGGATCCAGTGCATACCGCCAAAAG-3’), 2.5 U polymerase, and 0.4 mmol/l of dNTP mixture. The amplification conditions for long PCR were as follows: 95°C for 2 min, 30 cycles of denaturing (95°C for 15 s), annealing (62°C for 1 min), and extension (68°C for 9 min). The PCR products were run on 0.8% agarose gels and visualized by ethidium bromide staining. Then, the intensity of the 8.7 kb band was analyzed by Image J2x software.

### Annexin V-FITC/PI staining and flow cytometry analysis for apoptotic sperm detection

One of the apoptotic events occurs when the membrane phospholipid phosphatidylserine (PS) translocates from the inner to the outer leaflet of the plasma membrane. The PS translocation of sperm was determined by Annexin V staining in combination with propidium iodide (PI), which stains dead cells, according to the manufacturer’s instructions (BD Biosciences, San Jose, CA). In total, 2×10^6^ sperm cells were washed with PBS and were then resuspended in the binding buffer containing 2 μl of Annexin V-FITC and 2μl of PI for 10 min at room temperature in the dark. The samples were analyzed with a flow cytometer (Accuri C6, BD Biosciences, San Jose, CA). The sperm were classified as Annexin V^–^/PI^−^sperm (living cells), Annexin V^+^ sperm (apoptotic cells), or Annexin V^–^/PI^+^ sperm (necrotic cells), and the results were expressed as the percentage of total sperm.

### Statistical analysis

Basic information of the study population, conventional semen parameters, apoptotic markers, and mitochondrial biomarkers were described using untransformed data. Descriptive results were presented as the mean ± SD, No. (%), or percentiles (5th, 50th and 95th). Spearman correlation coefficients were used to explore associations of mitochondrial biomarkers with classic indicators of semen quality (conventional semen parameters and apoptotic parameters) and lifestyles. A standardized partial correlation analysis was further performed to assess relationships after adjusting for potential confounding variables. Multivariate linear regression models were used to analyze the associations of lifestyles with mtDNAcn and mtDNA integrity, both of which were log-transformed to better approximate the normality assumption of the model. The relationships between lifestyles and the dichotomized MMP via the 50th percentile were analyzed using non-conditional logistic regression models. As potential confounders, age, body mass index (BMI), duration of abstinence, tobacco smoking, and alcohol drinking were considered and included based on statistical and biologic considerations. The Statistical Package for the Social Sciences version 18.0 (SPSS, Chicago, IL, USA) was used for the statistical analysis in the current study.

## Results

### Population Characteristics

Basic characteristics of the study population are summarized in Table 1. A total of 666 participants attended the follow-up procedure in 2014. All of the subjects were undergraduate students with an average age of 21.4 years, a mean BMI of 21.6 kg/m^2^, and an average abstinence period of 4.2 days. The participants were also asked to report their smoking and drinking status. As Table 1 shows, approximately 22.1% of the subjects were current smokers and approximately three-fourths of the subjects were current drinkers.

**Table 1:** Characteristics of the study population (n = 666).

Characteristic	Values
Age (years)	21.4 ± 1.2
BMI (kg/m^2^)	21.6 ± 2.8
Abstinence duration (days)	4.2 ± 1.4
Tobacco smoking^a^	
Never	483 (72.5)
Quit	35 (5.3)
Current	147 (22.1)
Alcohol drinking	
Never	130 (19.5)
Quit	41 (6.2)
Current	495 (74.3)

BMI = body mass index.

Values are presented as the mean ± SD or no. (%).

^a^One subject failed to report tobacco smoking on 2014.

### Mitochondrial biomarkers and semen quality parameters

**Fig 1:**
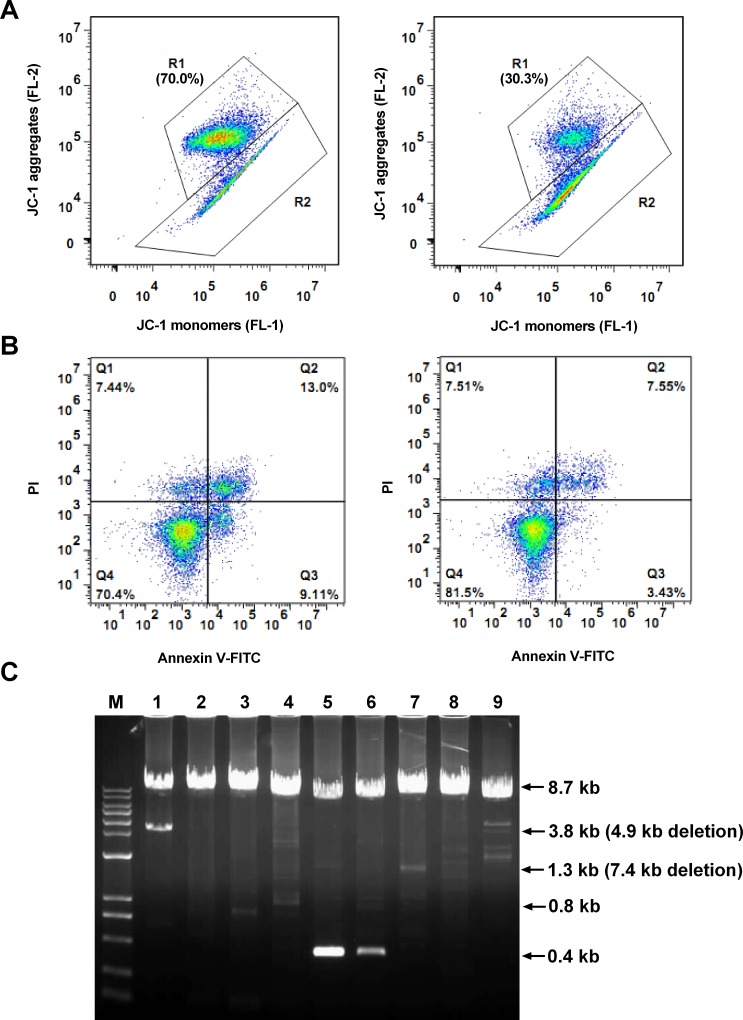
Representative measurements of sperm mitochondrial membrane potential (MMP), mtDNA integrity and apoptosis. **(A)** Scatter gates and dot plots of two representative sperm samples stained by JC-1. Region 1 (R1) shows the sperm subpopulation with high MMP and region 2 represents the subpopulation with low MMP. **(B)** Scatter gates and dot plots of two representative sperm samples stained by Annexin V/PI. The sperm were classified as normal viable (Annexin V^–^/PI^–^), apoptotic (Annexin V^+^), or necrotic (Annexin V^–^/PI^+^). **(C)** Long PCR product electrophoresis for mtDNA integrity. The 8.7-kb band represents wild-type full-length mtDNA, and the smaller bands represent deletions. Lane marker: 1-kb ladder plus DNA marker (Biomed, Beijing, China); lanes 1–9: nine representative PCR products after amplification from nine participants.

Fig 1A showed two representative semen samples with different levels of MMP detected by JC-1 staining and flow cytometry analysis. Region 1 (R1) represents the sperm subpopulation with high MMP, and R2 shows the subpopulation with low potential. The percentage of sperm cells in R1 was regarded as the MMP of each semen sample. Fig 1B showed that the Annexin V/PI staining classified sperm cells as the different subpopulations mentioned above. In addition, DNA of sperm cells was extracted and long PCR was next performed to detect the integrity of mtDNA. If the participant had some DNA fragmentations or deletions in this region of mtDNA, a decrease in the full-length PCR product would be found. As shown in Fig 1C, lane 2 indicated normal intact mtDNA, without any fragmentations or deletions; lanes 1, 5 and 6 showed relatively low amounts of full-length mtDNA band and obvious deletions, indicating poor mtDNA integrity. The other lanes (lanes 3, 4, 7, 8 and 9) also had slight deletions. To quantify mtDNA integrity, the intensity of the full-length (8.7 kb) band was measured and normalized to the mtDNAcn. Descriptive data of these mitochondrial biomarkers and classic indictors of semen quality were summarized in the Table 2. Because of various restrictions, such as detection time, multiple detection purposes that caused a lack of sperm cells for DNA extract, DNA quality and quantity, etc., the number of eligible value for MMP, mtDNAcn, mtDNA integrity, conventional semen parameter, and apoptosis were 627, 386, 362, 656 and 628 respectively. In the present study, 78% of all participants met the 2010 WHO criteria on conventional semen parameters. Among those participants with normal semen parameters, the 5th and 95th percentiles of MMP value were 51.5% and 87.4%, respectively.

**Table 2:** Distribution of sperm mitochondrial biomarkers and semen quality parameters.

Variables	Mean ± SD	Selected percentiles		
		5%	50%	95%
Mitochondrial biomarkers				
MMP, % (n = 627)	70.0 ± 13.7	43.8	73.0	86.7
mtDNAcn (n = 386)	3.68 ± 3.22	0.98	2.69	10.41
mtDNA integrity, AU (n = 362)	1052 ± 863	152	856	2772
Semen parameters (n = 656)				
Volume, ml	3.82 ± 1.89	1.74	3.56	6.53
Sperm concentration, 10^6^/ml	69.4 ± 61.2	13.7	51.8	194.3
Total sperm count, 10^6^	252.0 ± 221.0	42.6	193.3	707.9
Progressive motility (PR), %	55.4 ± 16.2	25.9	57.0	78.8
Apoptotic parameter (n = 628)				
Annexin V^+^ sperm, %	19.50 ± 7.96	9.54	17.84	34.87

MMP, mitochondrial membrane potential; MtDNAcn, mitochondrial DNA copy number; AU, arbitrary units.

### Correlation between mitochondrial biomarkers and semen parameters

**Fig 2:**
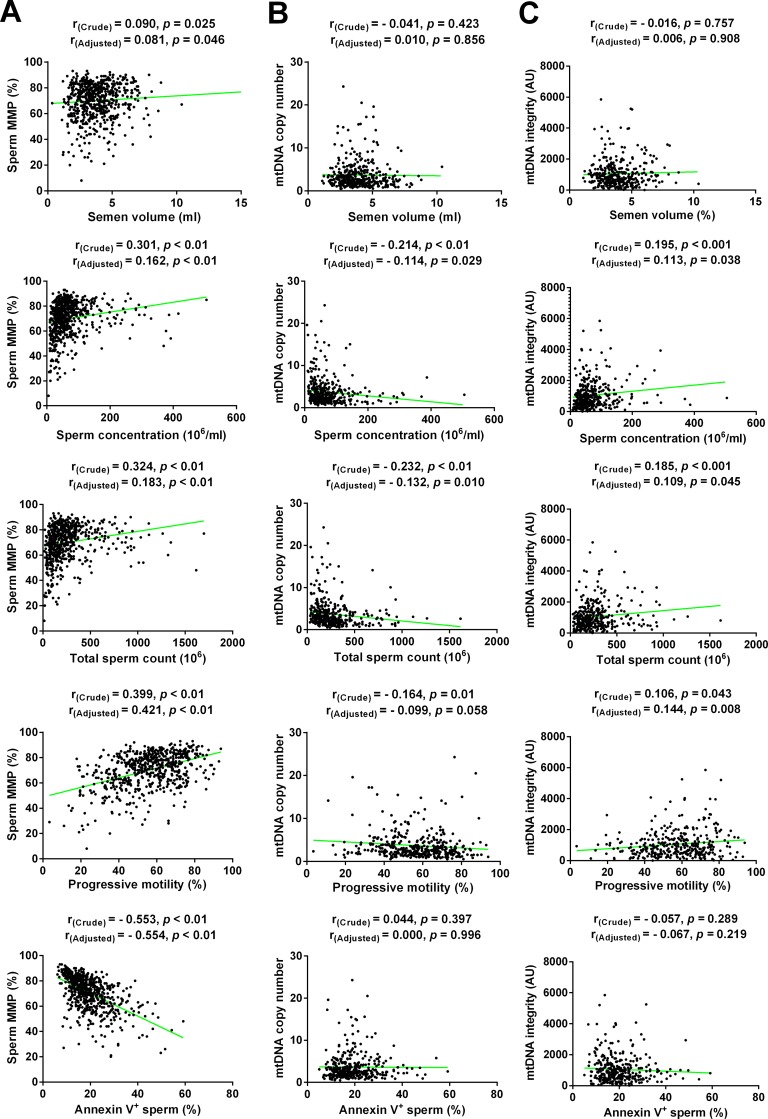
Association between mitochondrial markers and semen quality parameters. Scatterplots of semen quality parameters versus sperm mitochondrial membrane potential (MMP) **(A)**, mtDNA copy number **(B)**, and mtDNA integrity **(C)**. The crude correlation coefficients using Spearman’s correlation analysis and the adjusted coefficients using the standardized partial correlation analysis between mitochondrial biomarkers and semen quality parameters are shown.

We found that mitochondrial biomarkers were highly correlated with most of conventional semen parameters. As Fig 2 showed, MMP was significantly correlated with all of semen parameters including semen volume, sperm concentration, total sperm count, and progressive motility (r = 0.090, p = 0.025; r = 0.301, p<0.01; r = 0.324, p<0.01 and r = 0.399, p<0.01, respectively). mtDNA cn was significantly correlated with sperm concentration, total sperm count, and progressive motility (r = -0.214, p<0.01; r = -0.232, p<0.01 and r = -0.164, p = 0.01, respectively). mtDNA integrity was also significantly correlated with sperm concentration, total sperm count, and progressive motility (r = 0.195, p<0.01; r = 0.185, p<0.01 and r = 0.106, p = 0.043, respectively). Moreover, we found that MMP was significantly negatively correlated with apoptotic sperm, namely Annexin V^+^ sperm (r = -0.553, p<0.01). Based on their importance in the literature and biological plausibility, age, BMI, duration of abstinence, smoking status and drinking status were considered as confounders. After adjusting for these potential confounders, these associations of mitochondrial biomarkers with conventional semen parameters and apoptotic parameters remained.

### Effects of lifestyles on mitochondrial markers

We further explored the effects of population characteristics and lifestyles on these mitochondrial markers (Table 3). A nonparametric correlation revealed that drinking was significantly associated with a higher level of sperm MMP (p = 0.017); age was also correlated with increased mtDNAcn (p = 0.056). After adjustment for potential confounders, the relationship between dichotomized MMP and drinking status remained significant (OR = 1.31 [95% CI, 1.06, 1.60], p<0.05) (Table 4); mtDNA cn increased by an average of 9.35% [95% CI, 2.94, 16.2] per unit increase in age.

**Table 3:** Correlation coefficients of sperm mitochondrial biomarkers and lifestyles.

Characteristic and lifestyles	MMP, %		mtDNAcn		mtDNA integrity, AU	
	r	p-value	r	p-value	r	p-value
Age, years	-0.010	0.805	**0.097**	**0.056**	-0.063	0.234
BMI	0.030	0.448	0.004	0.936	0.012	0.819
Abstinent duration, days	0.012	0.772	-0.006	0.904	0.036	0.498
Tobacco smoking	0.070	0.081	-0.025	0.620	0.048	0.361
Alcohol drinking	**0.095**	0.017*	-0.041	0.417	0.002	0.973
Tea consumption	0.020	0.620	-0.022	0.664	0.025	0.636
Coffee consumption	-0.015	0.704	-0.040	0.437	-0.015	0.781
Cola consumption	0.059	0.143	0.036	0.478	-0.067	0.202
Fried foods consumption	-0.042	0.296	-0.002	0.966	-0.045	0.394
Baked foods consumption	0.042	0.299	0.046	0.366	-0.034	0.516
Tight-fitting underwear use	0.026	0.512	0.005	0.916	0.028	0.592
Chemical fiber underwear use	-0.056	0.226	-0.002	0.969	-0.018	0.774
Hot shower	-0.037	0.350	0.009	0.867	0.030	0.574
Sauna experience	-0.006	0.877	0.035	0.496	0.004	0.946
Bicycle riding	-0.026	0.519	-0.041	0.423	0.046	0.385
Motorcycle riding	-0.040	0.321	-0.063	0.213	0.085	0.107
Time of keeping seated	-0.030	0.455	-0.053	0.295	0.082	0.118

MMP, mitochondrial membrane potential; MtDNA cn, mitochondrial DNA copy number; AU, arbitrary units.

*P<0.05.

**Table 4:** Adjusted ORs or regression coefficients of mitochondrial biomarkers and lifestyles.

	MMP^a^, %	mtDNAcn^b^
Age	/	9.35 (2.94, 16.2)**
Alcohol drinking	1.31 (1.06, 1.60)*	/

MMP, mitochondrial membrane potential; mtDNAcn, mitochondrial DNA copy number.

^a^Dichotomized. The results were presented as ORs with the 95% confidence interval (CI).

^b^Log10-transformed. The results are presented as back-transformed regression coefficients (percentage change per 1-unit increase in the characteristic of the study population) with 95% CI.

Estimates were adjusted for age, BMI, abstinence duration, tobacco smoking, and alcohol drinking.

*P<0.05.

**P<0.01.

We found no associations between other lifestyles and mitochondrial markers. More detailed information about the lifestyle questionnaire can be found in the previous report [20].

## Discussion

Although the diagnostic capabilities of conventional semen parameters are improving, approximately 15% of men with normal basic semen analysis profiles are unexplainably infertile [5]. To adequately diagnose male infertility and to explore potential effects of low level of environmental exposures on male reproductive capacity, the search for optimal and mechanism-based predictors of semen quality becomes necessary and has resulted in a growing focus on these mitochondrial markers.

MMP is regarded as a major parameter that reflects mitochondrial functionality. It has been well demonstrated to be clearly correlated with conventional semen parameters such as sperm motility [21–23] and with fertilization ability in patients undergoing assisted reproduction [24]. However, the fact that these studies recruited participants from infertility clinics or assisted reproduction projects may limit the applicability of this relationship in the general population. Moreover, several studies suggest that Rh123 or DiOC_6_ (3) is not a reliable probe for analyzing MMP because of low sensitivity and the presence of several energy-independent probe binding sites in the mitochondria [21, 25]. In contrast, the fluorescent dye JC-1 has been increasingly applied for the detection of human sperm MMP [15, 26]. We therefore measured the level of MMP in the general study population using JC-1 staining. Moreover, the reduction in sperm MMP appears earlier in the apoptosis of mitochondrial pathway [16]. Abnormal sperm apoptosis and its related DNA fragmentation have been well demonstrated to have a prognostic power in male infertility and could serve as crucial indicators of semen quality [27–30]. Thus, we also investigated the relationship between sperm MMP and the apoptotic parameters detected by Annexin V/PI staining. In the present study, we found significant positive correlations between human sperm MMP and almost all of conventional semen parameters, including semen volume, sperm concentration, sperm count, and progressive motility. Furthermore, MMP was found to be negatively associated with the percentage of Annexin V^+^ sperm. The results indicated that the mitochondrial biomarker may serve as an important predictor of semen quality, as well as a predictor of the state of spermatogenesis in the general population.

One of the main characteristics of human sperm is motility, which is required to ensure male fertility. However, there is extensive debate regarding energy sources related to sperm motility: glycolysis or OXPHOS? [31]. Based on an analysis of a large cohort of men from the general population, these results provide direct evidence highlighting that the role of mitochondrial functionality in human sperm motility must not be underestimated.

Compared to nuclear DNA, no DNA repair system and some protective DNA-binding proteins provide the protection for mtDNA. Moreover, mtDNA is located near the inner mitochondrial membrane, where the main source of endogenous ROS is produced. Thus, mtDNA might be more susceptible than nuclear DNA to damage factors. However, the relationship between mtDNA integrity, one of major mitochondrial genetic features, and semen quality was inconclusive [32–35]. Therefore, we expected mtDNA integrity to be correlated with those classic indicators of semen quality in the general population. In the present study, mtDNA integrity was detected using long PCR based on the principle that mtDNA lesions can slow or block the progression of DNA polymerase, followed by normalization by mtDNAcn. We found that mtDNA integrity was associated with sperm concentration, sperm count, and progressive motility. The results demonstrated that both nuclear DNA integrity and mtDNA integrity are of great importance to the diagnosis of male infertility.

mtDNAcn is another major mitochondrial genetic features in addition to mtDNA integrity. We measured relative mtDNAcn using real-time PCR in each sperm sample, showing an average of 3.68 copies per spermatozoon, ranging from 0.52 to 24.28 copies. Furthermore, mtDNAcn was found to be negatively associated with most of conventional semen parameters, including sperm concentration, sperm count, and progressive motility. This also confirmed the previous reports of increased copy numbers of mtDNA in semen samples with abnormal parameters [17, 19]. Several reasons can explain these negative relationships. First, mtDNAcn decreases during normal spermatogenesis [36]. Abnormal spermatogenesis or aborted apoptosis can result in an increase in mtDNAcn in human sperm, as well as abnormal semen parameters [36]. Second, a feedback response compensating for defective mitochondria with mutated or fragmented mtDNA also results in an increase in mtDNAcn [37], but the compensation may fail to recover the mitochondrial dysfunction and normal spermatogenesis. Third, increased mtDNAcn has been associated with elevated oxidative stress [38–39]. ROS can cause abnormalities of sperm concentration, motility, morphology, and DNA integrity, resulting in difficulties in achieving pregnancy. It has been well reviewed that the levels of ROS increased in abnormal sperm [40]. Although further validation is still required, an elevated ROS level appears to somewhat justify the negative relationship between mtDNAcn and conventional semen parameters.

Further, we investigated the potential effects of population characteristics on these mitochondrial biomarkers. In the general college population, the current drinkers were found to be associated with a higher level of sperm MMP. Reports of detrimental effects of alcohol on conventional semen parameters were contradictory [41–43]. Moreover, there is no evidence, to our knowledge, regarding the effects of drinking status on sperm MMP in a population study. However, it has been suggested that alcohol consumption is associated with higher testosterone [44], which was demonstrated to restore the decline of sperm MMP induced by spinal cord injury [45]. Recently, an experimental study revealed that sub-lethal concentration of ethanol supplemented into semen extender could increase the sperm MMP, benefiting sperm cryopreservation [46]. Although, further validation is still necessary, this evidence appears to support the effect of drinking on sperm MMP in the present study. Additionally, mtDNAcn in sperm cells increased with age, which provides direct evidence why age should be adjusted for those studies regarding the effects of damage factors on sperm mtDNA cn. Semen quality decreases and oxidative stress increases with age [47–49]; thus, it is easy to understand why mtDNAcn in spermatozoa increases with human age.

There are some limitations to this study. First, the single semen sampling conducted during the interview likely introduced intraindividual variability of semen quality. Second, self-reported questionnaires might increase the possibility of information bias. Third, the age of these young men varies within a small scale, and these positive results need be further validated in other populations with different ages. In addition, the present study focused on total levels of mtDNA integrity, not on individual deletions; thus, Sanger sequencing of certain smaller PCR products was not performed. Nevertheless, the fact that the participants were recruited from the general student population and not from a typical infertility population made the study typical and relevant.

In conclusion, the strong correlations among MMP, mtDNAcn, mtDNA integrity and classic parameters of semen quality suggest that these mitochondrial biomarkers can reflect semen quality and serve as important indicators of spermatogenesis in the general population. These mechanism-based biomarkers combined with classic parameters of semen quality might be helpful for the diagnosis of male infertility. Furthermore, the present study provides novel clues for the effects of population characteristics and lifestyles on such mitochondrial markers.
